# A Human Neural Crest Stem Cell-Derived Dopaminergic Neuronal Model Recapitulates Biochemical Abnormalities in *GBA1* Mutation Carriers

**DOI:** 10.1016/j.stemcr.2017.01.011

**Published:** 2017-02-16

**Authors:** Shi-Yu Yang, Michelle Beavan, Kai-Yin Chau, Jan-Willem Taanman, Anthony H.V. Schapira

**Affiliations:** 1 Department of Clinical Neurosciences, UCL Institute of Neurology, Rowland Hill Street, London NW3 2PF, UK

**Keywords:** neural crest stem cells, Parkinson disease, glucocerebrosidase, α-synuclein, chaperone, PD modeling, dopaminergic neurons, *GBA1* mutation, ambroxol, *GBA1*-associated PD

## Abstract

Numerically the most important risk factor for the development of Parkinson's disease (PD) is the presence of mutations in the glucocerebrosidase *GBA1* gene. In vitro and in vivo studies show that *GBA1* mutations reduce glucocerebrosidase (GCase) activity and are associated with increased α-synuclein levels, reflecting similar changes seen in idiopathic PD brain. We have developed a neural crest stem cell-derived dopaminergic neuronal model that recapitulates biochemical abnormalities in *GBA1* mutation-associated PD. Cells showed reduced GCase protein and activity, impaired macroautophagy, and increased α-synuclein levels. Advantages of this approach include easy access to stem cells, no requirement to reprogram, and retention of the intact host genome. Treatment with a GCase chaperone increased GCase protein levels and activity, rescued the autophagic defects, and decreased α-synuclein levels. These results provide the basis for further investigation of GCase chaperones or similar drugs to slow the progression of PD.

## Highlights

• A neuronal model recapitulates biochemical abnormalities in *GBA1* mutation PD
• Ambroxol rescued the autophagic defects in human *GBA1* mutant neuronal cells
• Ambroxol decreased α-synuclein levels human *GBA1* mutant neuronal cells

## Teaser

Schapira and colleagues devised a dopaminergic neuronal model from human adipose NCSCs which provided insights into the role of glucocerebrosidase dysfunction in PD pathogenesis. Glucocerebrosidase function enhancement reversed biochemical abnormalities in GBA1 mutant neurons. This method provides a platform to test potential therapeutic compounds for PD treatment.

## Introduction

Parkinson's disease (PD) is a progressive neurodegenerative disorder currently affecting 4% of the population >80 years and whose prevalence is expected to double by 2030 (de Lau and Breteler, 2006). Multiple neurotransmitter systems are involved in the neurodegeneration of PD, but the loss of substantia nigra dopaminergic neurons is responsible for the dominant early motor features. The pathological hallmark of PD is the accumulation and aggregation of α-synuclein and the deposition of Lewy bodies. A heterogeneous range of etiologically and pathogenically relevant factors have been identified for PD, and it is likely that the neurodegeneration and clinical manifestations of the disease are the end result of multiple aberrant pathways (Schapira and Jenner, 2011). Several single gene defects have been identified in familial and apparently sporadic PD (Volta et al., 2015). Numerically the most important genetic risk factor for PD is the presence of mutations of the glucocerebrosidase gene *GBA1* (Sidransky et al., 2009). Although precise estimates vary between populations, approximately 5%–10% of PD patients carry *GBA1* mutations, their presence increasing the risk for PD in any one individual by 20–30 times (Beavan and Schapira, 2013).

Homozygous *GBA1* mutations are the cause of the autosomal recessive lysosomal storage disorder Gaucher disease (GD). Both homozygous and heterozygous *GBA1* mutation carriers have a similar risk for the development of PD in later life, although onset in GD patients may be earlier (Alcalay et al., 2014). The PD expressed in *GBA1*-positive patients is clinically indistinguishable from sporadic PD, except for slightly earlier onset and more cognitive dysfunction. Pharmacological responses are identical, imaging and pathology being the same as for sporadic PD (Schapira, 2015).

Interest has focused on the molecular mechanisms by which *GBA1* mutations and reduced activity of the glucocerebrosidase enzyme (GCase) increase the risk for PD. A reciprocal relationship between GCase activity and α-synuclein levels has emerged as an important candidate that may influence the development and progression of PD pathology (Sardi et al., 2015). There are several potential processes by which reduced GCase activity may result in increased α-synuclein levels and vice versa, including GCase trafficking defects, lysosomal dysfunction, substrate accumulation, and disordered lipid membrane function (Siebert et al., 2014).

To investigate further biochemical effects of *GBA1* mutations, we have developed dopaminergic neuronal lines from neural crest stem cells (NCSCs) obtained from PD and *GBA1* mutation subjects. This model has also been used to examine the potential to manipulate the GCase/α-synuclein interaction to provide candidate molecules for further investigation as disease-modifying therapies in PD. We demonstrate that these patient-derived dopaminergic cells recapitulate the main biochemical abnormalities seen in PD postmortem brain and that through the use of a small-molecule chaperone, GCase activity can be increased and α-synuclein levels reduced.

## Results

### Isolation and Characterization of Human Adipose Neural Crest Stem Cells

**Figure 1:**
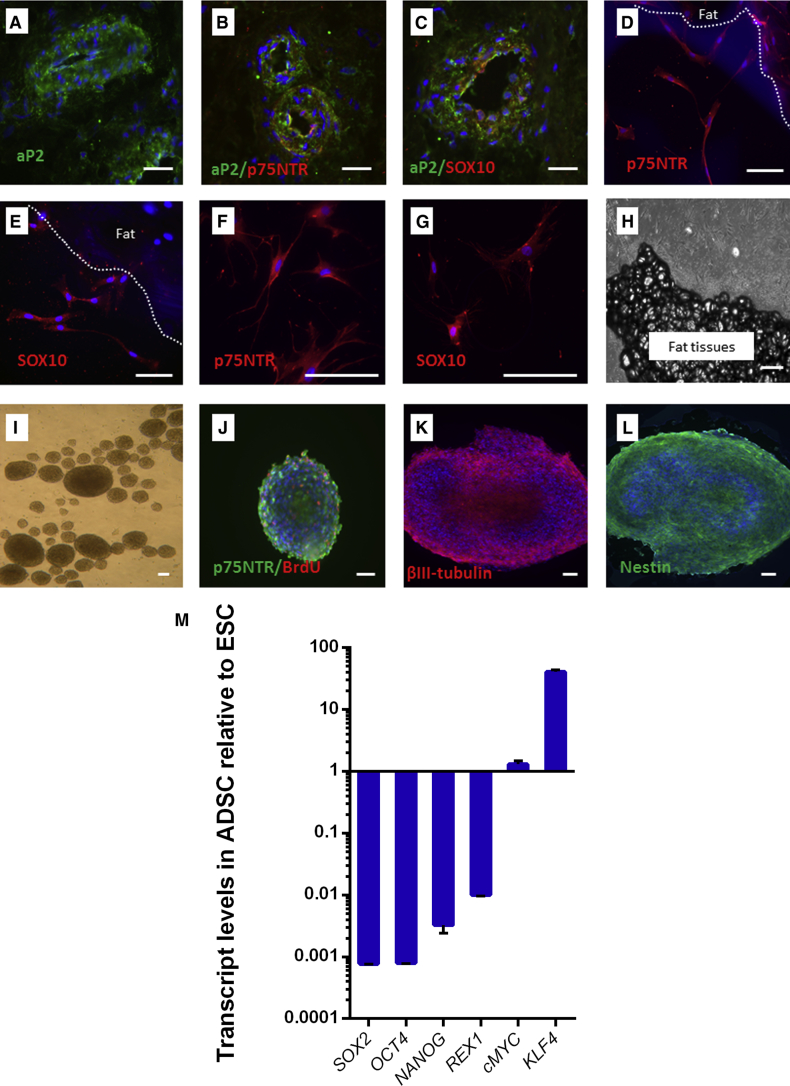
Identification, Isolation, and Characterization of Neural Crest Stem Cells from Human Adipose Tissues (A) An anti-adipose protein 2 (aP2) antibody (green) was used to identify stem cell niches in human fat tissue. (B and C) Two antibodies (anti-p75NTR and SOX10, both red) were employed for co-immunostaining with the anti-aP2 antibody in human adipose tissues, which revealed that some cells express p75NTR and SOX10 in the human adipose stem cell niche. (D and E) Three to five days after explanting, human adipose tissue showed p75NTR- and SOX10-expressing cells (both red) migrating from the tissue. (F and G) Migrated cells were cultured and maintained under feeder-free conditions for more than 18 months with more than 24 cell passages and still expressed p75NTR and SOX10 (both red). (H) Three-week-old human adipose explant showing numerous cells that migrated from the tissue. (I) Neurospheres formed after 6 days of culturing in neurosphere formation medium. (J) The cell proliferation marker BrdU (red). (K and L) The neuronal marker β-III tubulin (red) and the neuronal progenitor maker nestin (green) were expressed in the neurospheres. Nuclei were stained with DAPI (blue). (M) The pluripotent gene expression in haNCSC was examined by real-time qRT-PCR and compared with human ESC line (SHEF-6). ADSC, adipose-derived stem cells. Relative expression values are plotted as means ± SEM from three independent experiments (n = 3). Scale bars, 50 μm.

Anti-aP2 antibody immunostaining was used to localize stem cell niches in blood vessel walls of human adipose tissues (Figure 1A), a feature consistent with previous observations (Shan et al., 2013). Anti-p75 neurotrophin receptor (p75NTR) and anti-SOX10 (SOX10) antibodies were used to confirm that adipose stem cell niches contained NCSCs (Figures 1B and 1C). p75NTR is a nerve growth factor receptor highly expressed in NCSCs, and has been used to isolate NCSCs from neural tube in embryonic day 10 (E10) and E9 mice (Stemple and Anderson, 1992); SOX10 is a member of a transcription factor family (SRY-related HMG box) involved in the determination of NCSC fate and is highly expressed in NCSCs (Kim et al., 2003).

We explanted fresh human fat biopsy samples onto a 6-well plate pre-coated with fibronectin and provided energy-rich medium to enhance stem cell migration. After 3–5 days of explantation, the stem cells continuing to express p75NTR and SOX10 migrated from fat tissue (Figures 1D and 1E). After 3–4 weeks of explantation (Figure 1H), the migrated stem cells were detached using enzymatic methods, passaged, and cryopreserved. Human adipose NCSCs (haNCSCs), unlike their animal counterparts, are able to regenerate ex vivo under feeder-free conditions (Thomas et al., 2008). We cultured and maintained the stem cells under feeder-free conditions for more than 18 months with over 24 cell passages and confirmed continuing p75NTR and SOX10 expression (Figures 1F and 1G).

The ability to form neurospheres has been used to test ontogeny and multipotency of NCSCs (Nagoshi et al., 2008). haNCSCs from a range of passages were cultured in a serum-free sphere-forming medium supplemented with human epidermal growth factor, human fibroblast growth factor 2, and B27 supplement. Cells were maintained in the same medium for a further 6 days. Multiple neurospheres were formed (Figure 1I) and expressed the cell proliferation marker 5-bromo-2′-deoxyuridine (BrdU), neural progenitor maker nestin, immature neuron marker β-III tubulin, and neural crest cell marker p75NTR (Figures 1J–1L).

The pluripotent gene expression profile is varied depending on which stage the haNCSCs were isolated. If haNCSCs are isolated from the pre-migratory state in embryos, the gene expression profile is highly similar to that of human embryonic stem cells (ESCs) (Thomas et al., 2008). When haNCSCs are isolated from adult human neural crest-derived tissues, the expression of pluripotent stem genes are substantially different to human ESCs (Hauser et al., 2012). We used real-time qRT-PCR to examine the expression of six main pluripotent genes, *OCT4*, *SOX2*, *NANOG*, *REX*, *cMyc*, and *KLF4*, in haNCSCs; and compared their expression with those in a human ESC line (SHEF-6). We found that *OCT4*, *SOX2*, *NANOG*, and *REX1* are expressed at lower levels in haNCSCs compared with that in human ESCs, in agreement with previous studies (Hauser et al., 2012), while *cMyc* was expressed at the same level of human ESCs and *KLF4* was expressed at a higher level compared with human ESCs (Figure 1M). *KLF4* is a transcriptional regulator of genes critical for epithelial-mesenchymal transition (Tiwari et al., 2013), which is pre-step of neural crest cells migrating to various elected locations during embryo development (Nagoshi et al., 2008). As we used the migration procedure to isolate haNCSCs, the higher level of *KLF4* in haNCSCs than ESCs is to be expected.

A recent study comparing regulatory programs between neural crest cells and early ESCs found that neural crest cells retain core pluripotency genes (Buitrago-Delgado et al., 2015). We therefore examined the expression of the pluripotent genes *NANOG*, *OCT4*, *SSEA4*, and *TRA-1-81*, and the neural crest genes *p75NTR* and *SOX10*. HaNCSCs were isolated from age-matched controls, individuals with heterozygous *GBA1* (N370S/WT) mutations, and sporadic PD patients (for details see Table S1). The results showed that pluripotent and neural crest genes were expressed in all haNCSC lines, indicating that they retain neural crest cell features and pluripotent properties (Figures S1A–S1C). There were no differences in the expression of these genes between the haNCSC lines. Sonic hedgehog (SHH) is a signaling molecule that has been found to be involved in the development of early neural crest progenitors (Brito et al., 2006, Wada et al., 2005) and promotes the formation of multipotent progenitors endowed with both neural-melanocytic and mesenchymal differentiation potential (Calloni et al., 2007, Le Douarin et al., 2004). SHH executes its functions through a cell-surface transmembrane protein called Patched (PTCH) and a membrane-spanning receptor called Smoothened (SMO). We therefore examined the expression of PTCH and SMO in three groups of haNCSCs with flow cytometry analysis. More than 60% of cells stained positive with both PTCH and SMO proteins (Figure 1D), and there were no significant differences in the levels of these proteins between three haNCSC groups. These data support the conclusion that adult human adipose tissue contains a cell population that retains neural crest cell features and can be isolated with a simple migration protocol.

### Dopaminergic Neuronal Differentiation of haNCSCs

**Figure 2:**
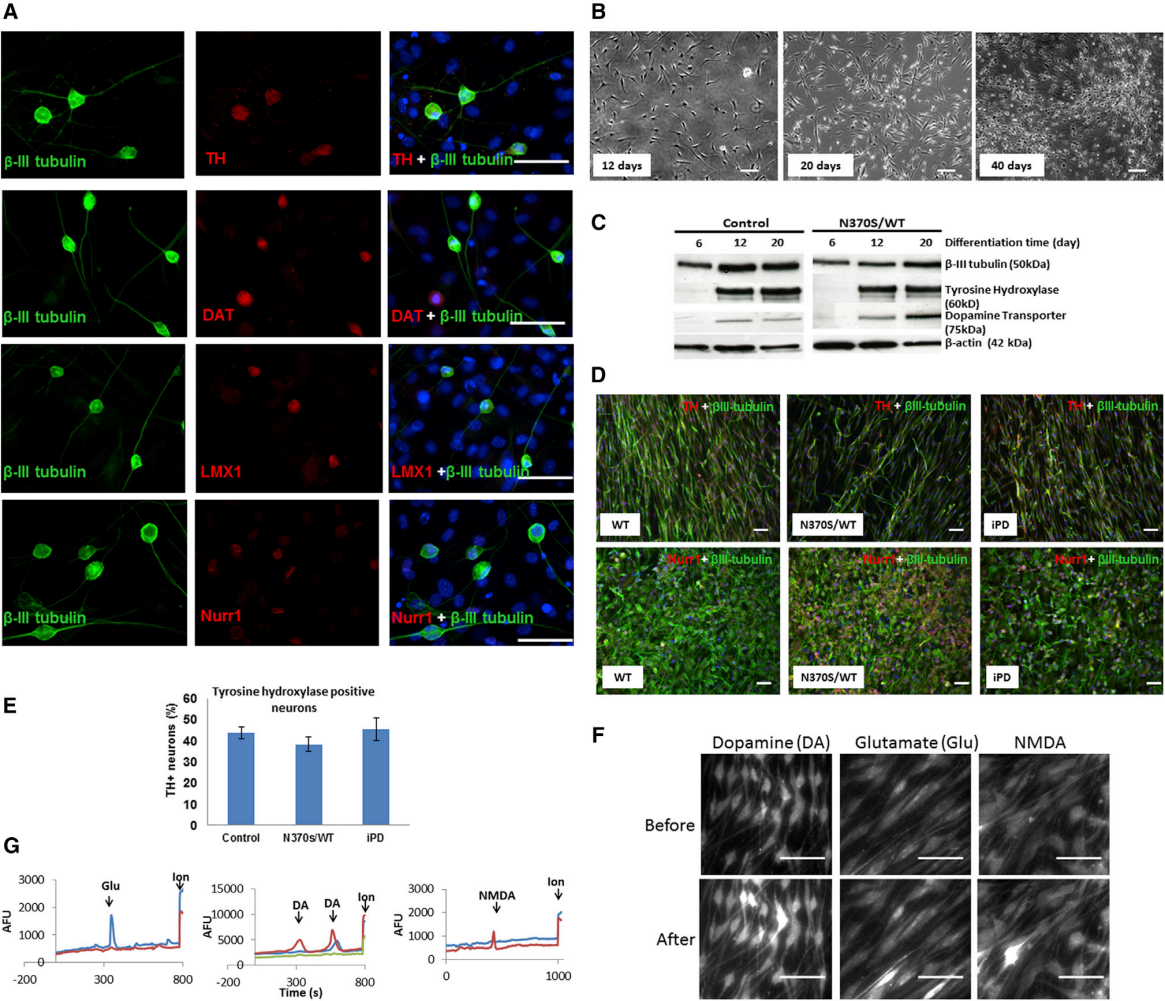
Dopaminergic Neuronal Differentiation of haNCSCs (A) The mouse PA6 cell line was used for dopaminergic neuronal differentiation via a co-culture procedure. After co-culturing for 40 days, human adipose neural crest stem cells expressed the dopaminergic neuronal makers tyrosine hydroxylase (TH, red) and the dopamine transporter (DAT, red), the midbrain markers LIM-homeodomain transcription factor (LMX1, red), and nuclear receptor-related protein 1 (Nurr1, red) and the pan-neuronal marker β-III tubulin (green). (B) When a defined program was used to differentiate adipose neural crest stem cells into dopaminergic neurons, the cells gradually changed their morphology toward neuron-like cells. (C) Western blot experiments showed that pan-neuronal (β-III tubulin) and dopaminergic neuronal makers (TH, DAT) were expressed after 20 days of defined neuronal differentiation. (D and E) After 40 days of defined neuronal differentiation, the percentage of TH^+^ neurons was 38%–52%. The proportion of TH^+^ neurons derived from wild-type (WT) control, heterozygous mutant *GBA1*, and iPD subjects was not significantly different. For all three groups n = 3 cell lines, and data are represented as mean ± SEM. (F and G) The expression of functional ligand-gated channels in differentiated neurons was examined by measuring cytosolic Ca^2+^ changes (bright) upon application of the neural transmitters (glutamate, dopamine, and NMDA) in cells loaded with Fluo-4 AM or Fura-2 AM. After 40 days of defined neuronal differentiation, up to 56% of cells responded to dopamine (DA), 21% responded to glutamate (Glu), and 16% responded to NMDA. Nuclei were stained with DAPI (blue). AFU, arbitrary fluorescence units. Scale bars, 50 μm.

A mouse stromal cell (PA6) line has been shown to be able to induce ESCs to form midbrain dopaminergic neurons (Kawasaki et al., 2000). We therefore initially used PA6 cells for haNCSC dopaminergic neuronal differentiation via a co-culture procedure. After co-culturing with a PA6 cell line for 40 days, haNCSCs were successfully induced to dopaminergic neurons, which express midbrain dopaminergic neuronal makers such as tyrosine hydroxylase (TH) (Figure 2A), dopamine transporter (DAT) (Figure 2A), LIM-homeobox transcription factor 1 (LMX1) (Figure 2A), nuclear receptor-related protein 1 (NURR1) (Figure 2A), and a general neuronal marker, β-III tubulin (Figure 2A). However, to generate more pure dopaminergic haNCSC cultures, we used a defined dopaminergic neuronal differentiation program proven to be able to generate midbrain dopaminergic neurons from human mesenchymal stem cells (Trzaska and Rameshwar, 2011). During the differentiation cells change their morphology toward neuron-like cells (Figure 2B), and express the neuronal marker β-III tubulin after 6 days (Figure 2C) and the dopaminergic neuronal makers TH and DAT after 12 days (Figure 2C). Following 40 days differentiation, the neurons become more mature and express both dopaminergic neuronal markers TH and midbrain neuronal markers NURR1 (Figure 2D). The proportion of dopaminergic neurons (TH^+^) derived from control, heterozygous mutant *GBA1*, and PD subjects was not significantly different (Figures 2D and 2E). The percentage of TH^+^ neurons was 38%–52% (Figure 2E).

To examine the maturation and function of neuronal cells, we examined the expression of functional ligand-gated channels in differentiated neurons by measuring intracellular Ca^2+^ changes upon application of the neuronal transmitters (glutamate, dopamine, and N-methyl-D-aspartate [NMDA]) in cells loaded with Fluo-4 AM or Fura-2 AM. After 40 days of neuronal differentiation, up to 56% of cells responded to dopamine and 21% responded to glutamate (Figures 2F and 2G), indicating that haNCSCs can be differentiated to functional dopaminergic neuronal cells.

### Establishment of haNCSC-Derived Neuronal Models of *GBA1*-Associated PD

**Figure 3:**
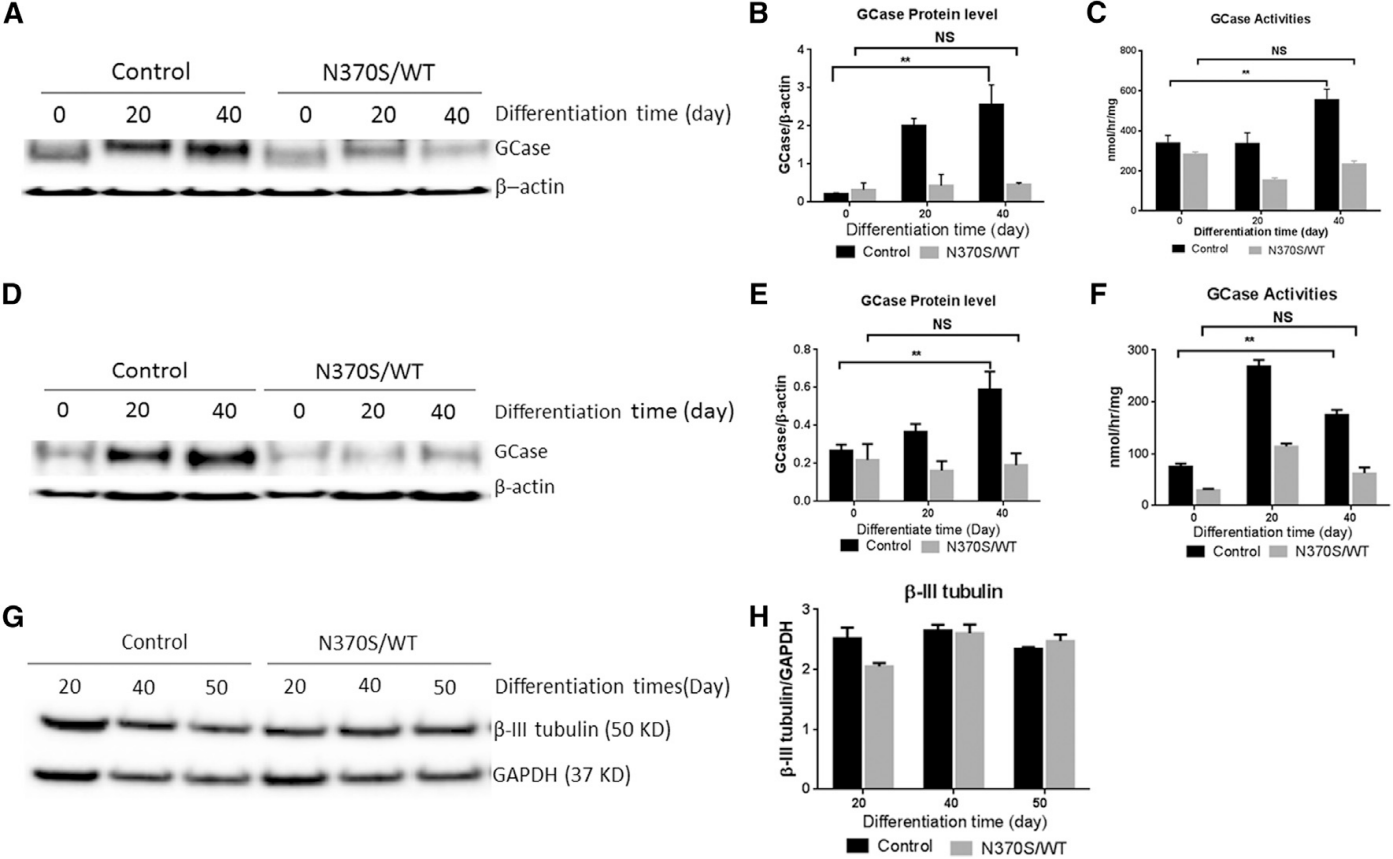
Establishment of haNCSC-Derived Neuronal Models of *GBA1*-Associated PD (A and B) Control and N370S/WT haNCSC lines were differentiated to dopaminergic neurons with the defined program. The GCase protein levels increased significantly in control haNCSC-derived neurons during differentiation, but were not increased in *GBA1* mutant (N370S/WT) haNCSC-derived neurons. (C) The activity of GCase also increased significantly in control haNCSC-derived neurons during differentiation, but this increase was not seen in *GBA1* mutant (N370S/WT) haNCSC-derived neurons. (D–F) Likewise, the GCase protein levels and activity in the iPSC-derived neurons increased significantly during differentiation in control subjects but not in subjects heterozygous for the *GBA1* mutation. (G and H) By examining the neuronal marker β-III tubulin in the differentiated cells, it was found that 40–50 days of differentiation is the optimal time window to examine the effects of therapeutic interference. Data are presented as means ± SEM from three independent experiments (n = 3). ^∗∗^p < 0.01; NS, not significant (p > 0.05). All comparisons were carried out with one-way ANOVA and Student's t test.

To investigate the effects of heterozygous *GBA1* mutations on GCase activity and protein levels in neuronal cells, we differentiated haNCSC cell lines from wild-type (WT; control) and heterozygous *GBA1* N370S/WT mutation (Table S1) to dopaminergic neuronal cells using the program described above. The cells were harvested at different stages (0, 20, and 40 days) of the differentiation program. The GCase activity was measured with a fluorogenic assay, and GCase protein level was determined by western blotting. The results showed that both the protein level and activity of GCase increased significantly in the WT stem cell-derived neurons compared with undifferentiated stem cells during differentiation (Figures 3A–3C). These increases did not occur in *GBA1* mutant (N370S/WT) haNCSC-derived neurons (Figures 3A–3C).

Induced pluripotent stem cell (iPSC)-derived neurons from *GBA1* mutation patients show reduced GCase enzymatic activity and protein levels (Schondorf et al., 2014). To validate and compare the abnormality seen in the haNCSC-derived heterozygous *GBA1* mutant neurons, we generated iPSCs from fibroblasts from control subjects and subjects heterozygous for the N370S *GBA1* mutation (Okita et al., 2011). The iPSC clones from both control and mutant subjects expressed pluripotent genes (*NANOG*, *SSEA4*, *TRA-1-81*, and *OCT4*), and the expression of the pluripotent genes between control and *GBA1* mutation subjects was not significantly different (Figure S2A). Karyotyping results revealed that no translocations had occurred during cloning of iPSC (Figure S2C). The iPSC stem cells were differentiated into dopaminergic neurons expressing the neuronal marker (β-III tubulin) and dopaminergic neuronal markers TH and Nurr1 (Figure S2B). Although the pluripotent properties of iPSC clones were not significantly different between control subjects and subjects heterozygous for the *GBA1* mutation, the GCase activity and protein level were significantly reduced in neurons derived from subjects heterozygous for the *GBA1* mutation differentiation (Figures 3D–3F). These results are not only consistent with the previous iPSC studies, but also confirm the abnormality seen in the haNCSC-derived heterozygous *GBA1* mutant neurons (Figures 3A–3C). The level of β-III tubulin expression in the haNCSC-derived neurons is constant at 40–50 days of differentiation, suggesting that the neuronal cell population is stable at this time in both control and *GBA1* mutation subjects (Figures 3G and 3H). We therefore selected this time period to examine the effects of therapeutic intervention with ambroxol.

### Heterozygous *GBA1* Mutations Decrease GCase Activity and Protein Levels in Human Neurons

**Figure 4:**
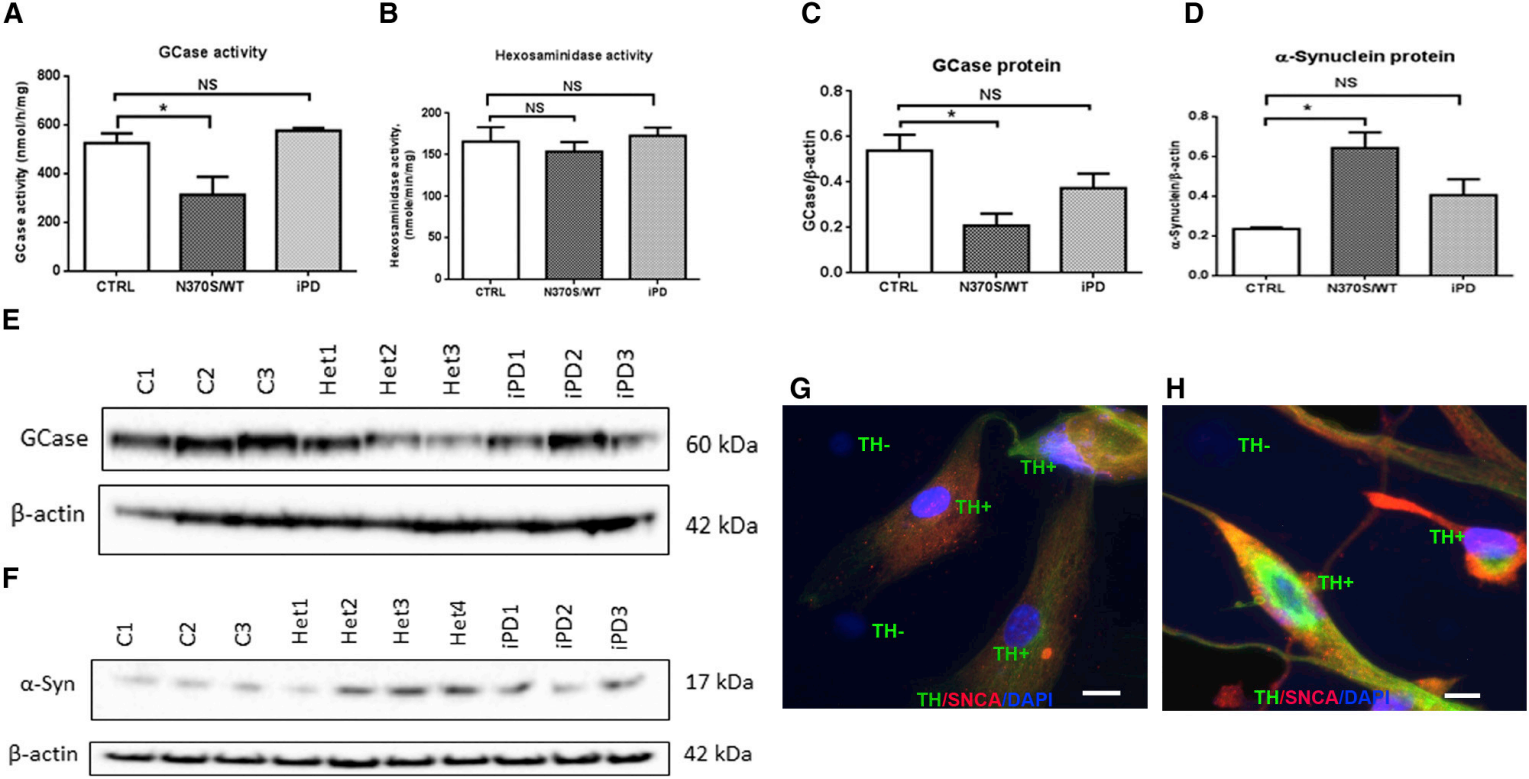
*GBA1* Mutation Decreases GCase Activity and Protein Levels and Increases α-Synuclein in haNCSC-Derived Dopaminergic Neurons (A, C, and E) HaNCSCs derived from controls (CTRL, C1–3), carriers of the heterozygous *GBA1* mutation (N370S/wt; Het1–3), and idiopathic Parkinson's disease (iPD1–3) patients were differentiated into dopaminergic neurons for 40 days. GCase activity and the level of GCase protein were significantly lower in the *GBA1* mutation group, but not in the iPD group, compared with the control group. For all three groups n = 3 cell lines; data are represented as mean ± SEM. (B) Hexosaminidase, another lysosomal enzyme, was not affected by the *GBA1* mutation. For all three groups n = 3 cell lines; data are represented as means ± SEM. (D and F) The level of α-synuclein was significantly increased in the *GBA1* mutation group, but not in iPD group, compared with the control group. For both control and iPD groups n = 3 cell lines; for the *GBA1* mutation group (Het1–4) n = 4 cell lines; data are represented as mean ± SEM. (G and H) The level of α-synuclein protein (red) was identified in the untreated *GBA1* mutant (N370S/WT) human neurons. Results indicate that dopaminergic neurons (TH^+^, green) have a higher level of α-synuclein protein. Nuclei were stained with DAPI (blue). All comparisons were carried out with one-way ANOVA and Student’s t test. ^∗^p < 0.05; NS, not significant (p > 0.05). Scale bars, 10 μm.

A recent study using iPSCs with homozygous *GBA1* mutations described widespread lysosomal depletion (Awad et al., 2015). To investigate the effects of heterozygous *GBA1* mutations on GCase activity, we differentiated haNCSCs derived from controls and subjects heterozygous for the *GBA1* mutation (N370S/WT) and idiopathic PD (iPD) into dopaminergic neurons with the established differentiation program for 40 days. GCase activity was significantly reduced by 40.2% (p = 0.0307) in the *GBA1* mutation group compared with control. GCase activity was not reduced in the iPD-derived neurons (Figure 4A). Hexosaminidase activity, another lysosomal enzyme, was not significantly different in the different cell lines (Figure 4B). The GCase protein level in N370S/WT stem cell-derived neurons was significantly decreased by 61.3% (p = 0.0257) compared with WT (Figures 4C and 4E), consistent with previous iPSC-derived neuronal studies (Schondorf et al., 2014). There was a trend for the GCase protein level in idiopathic PD stem cell-derived neurons to be decreased, but this was not statistically significant.

### *GBA1* Mutation Increased α-Synuclein Level in haNCSC-Derived Dopaminergic Neurons

Inhibition of GCase led to the accumulation of α-synuclein in in vitro and in vivo models (Cleeter et al., 2013, Manning-Bog et al., 2009). The iPSC-derived neuronal model from both GD and heterozygous *GBA1* mutant demonstrated accumulation of α-synuclein (Mazzulli et al., 2011). In our model, the level of α-synuclein was significantly increased by 172% (p = 0.02) in the neurons derived from *GBA1* mutation haNCSC-derived dopaminergic neurons compared with controls (Figures 4D and 4F). Immunocytochemistry in the GBA1 mutant (N370S/WT) human neurons showed that the level of α-synuclein protein was higher in the dopaminergic as opposed to the non-dopaminergic cells in culture (Figures 4G and 4H). There was a trend for the level of α-synuclein in the idiopathic PD group to be elevated, but this did not reach statistical significance (Figures 4D and 4F).

### *GBA1* Mutation Impaired Macroautophagy in haNCSC-Derived Dopaminergic Neurons

**Figure 5:**
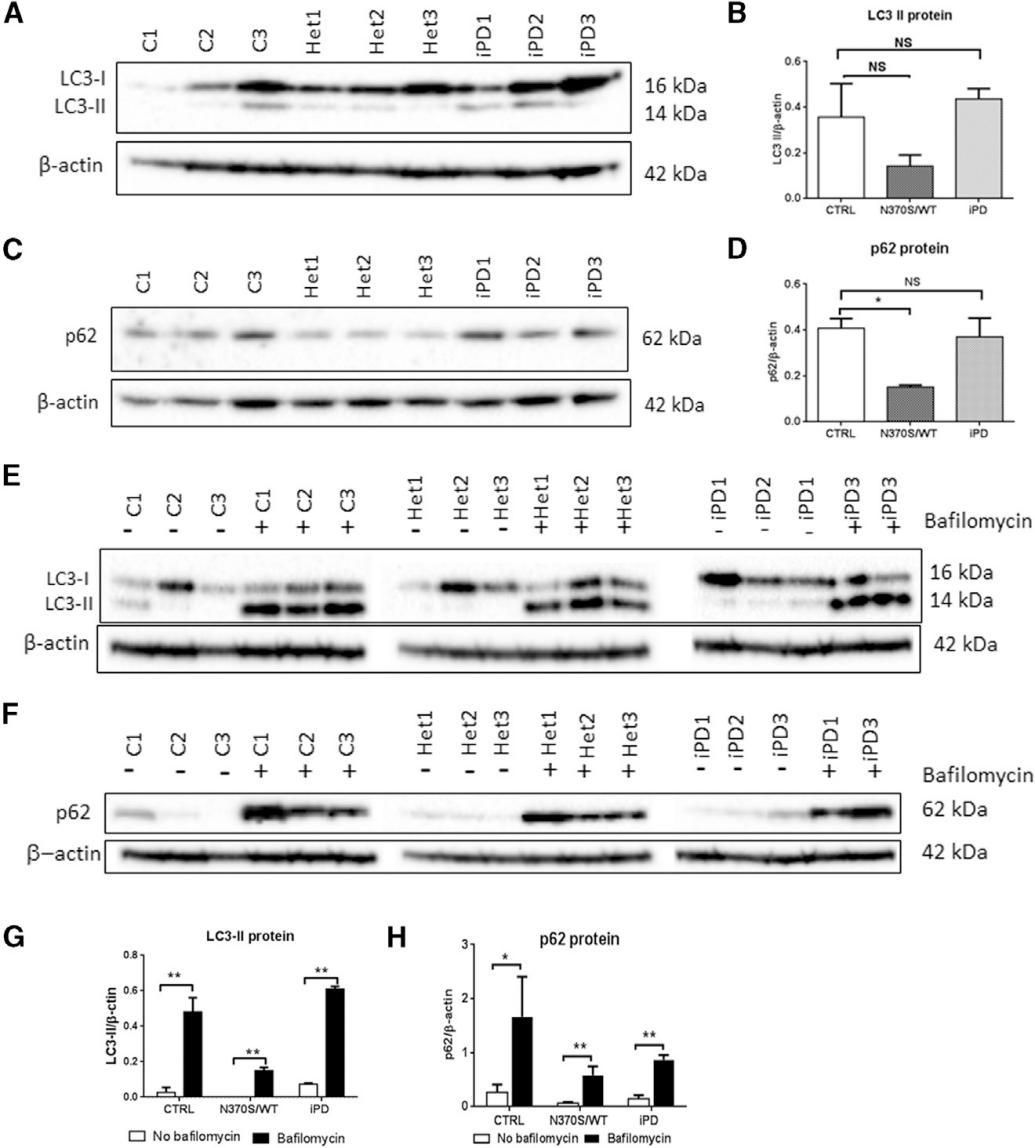
HaNCSC-Derived Dopaminergic Neurons from *GBA1* Mutant Carriers Show Defective Lysosomal Autophagy (A and B) LC3-II appeared decreased in the *GBA1* mutation group (N370/wt; Het1–3) compared with the control (C1–3; CTRL) and iPD (PD1–3) groups, but the decrease was not statistically significant. (C and D)The ubiquitin-associated protein p62 was significantly decreased in the *GBA1* mutation group compared with the other two groups. (E–H) When the neurons were incubated with the inhibitor of autophagy bafilomycin, the LC3-II and p62 level increased significantly, indicating that the fusion of lysosomes with autophagosome was normal but the autophagosome number was reduced by the *GBA1* mutation; i.e., the creation of autophagosomes in the *GBA1* mutant neurons was decreased. For all three groups n = 3 cell lines; data are presented as means ± SEM. All comparisons were carried out with one-way ANOVA and Student's t test. ^∗∗^p < 0.01; ^∗^p < 0.05; NS, not significant (p > 0.05).

Postmortem PD brain (Murphy et al., 2014) and an iPSC-derived neuronal model from *GBA1* mutant PD patients (Schondorf et al., 2014) suggested that GCase deficiency may cause general autophagic dysfunction and disruption of the autophagy-lysosome pathway. We found that basal levels of light chain type 3-II protein (LC3-II), a marker of autophagosomes, were decreased, but not significantly so, in *GBA1* mutation-positive carrier neurons compared with control neurons (Figures 5A and 5B). The decrease of LC3-II in *GBA1* mutation-positive carrier neurons suggests, but does not confirm, that the N370S *GBA1* mutation impairs autophagosome formation.

P62 is a ubiquitin-binding scaffold protein acting as a cargo receptor recruited to the autophagosome membrane through interaction with LC3-II (Itakura and Mizushima, 2011), and is an alternative marker for detecting autophagic flux (Bjorkoy et al., 2005). P62 levels were decreased 62% (p = 0.0162) in *GBA1* mutant neurons (Figures 5C and 5D). To examine whether the decrease in LC3-II and p62 is due to a defect of the fusion of autophagosomes with lysosomes or to dysfunction of autophagosome formation, we inhibited the macroautophagy pathway at the autophagosome-lysosome fusion stage with bafilomycin. The results (Figures 5E–5H) showed that LC3-II and p62 protein levels were increased significantly after bafilomycin treatment. This indicates that the autophagosome-lysosome fusion pathway was normal and that the decrease in LC3-II and p62 protein levels in *GBA1* mutation neuronal cells are most likely due to impaired autophagosome formation.

### *GBA1* Mutation Does Not Affect LAMP2a or LAMP1

There was no significant change in levels of the chaperone-mediated autophagy (CMA) protein LAMP2a or LAMP1 in *GBA1* mutation carrier neurons when compared with control cells (Figures S3A–S3D).

### Ambroxol Treatment Increases GCase Activity and Protein Levels

**Figure 6:**
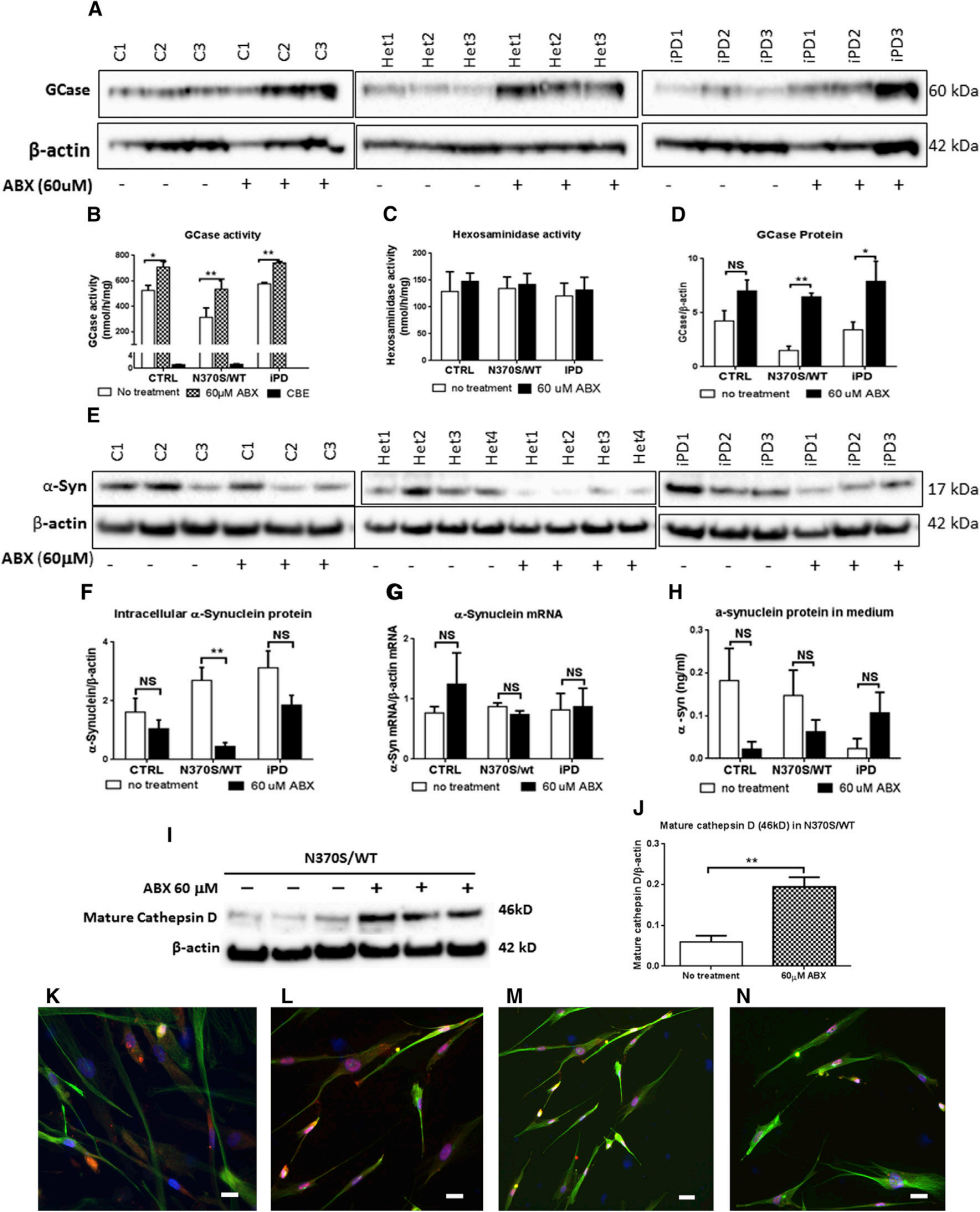
Ambroxol Treatment Recovers GCase Activity and Increases GCase Protein (A and D) The level of GCase protein was increased following ambroxol (ABX) treatment in heterozygous *GBA1* mutant (Het1–3; N370S/wt) and iPD (PD1–3) neurons, but not in neurons derived from controls (C1–3; CTRL). For all three groups n = 3 cell lines. (B) Ambroxol treatment significantly increased GCase activity in all three groups of neurons. Addition of the GCase inhibitor, conduritol-B-epoxide (CBE), showed that the contribution to the increase from non-lysosomal GCase (*GBA2*) was minimal. For all three groups n = 3 cell lines. (C)The activity of hexosaminidase was not affected by ambroxol treatment. For all three groups n = 3 cell lines. (E and F) Ambroxol treatment (60 μM) significantly decreased α-synuclein protein in *GBA1* mutant (N370S/WT) neurons. The lower level of α-synuclein protein also occurred in treated WT and iPD neurons, but the decrease was not significant. For control and iPD groups n = 3 cell lines; for the *GBA1* mutant group n = 4 cell lines. (G) The α-synuclein mRNA level in all three groups of neurons was not affected by ambroxol treatment. For control and iPD groups n = 3 cell lines; for *GBA1* mutant group n = 4 cell lines. (H) There was no significant difference in the amount of α-synuclein released in the culture medium between treated and untreated cells of all three groups. For control and iPD groups n = 3 cell lines; for *GBA1* mutant group n = 4 cell lines. (I and J) Mature cathepsin D protein level was significantly increased following ambroxol treatment in *GBA1* mutant (N370S/WT) human neurons. For all groups n = 3 cell lines. (K and L) High level of α-synuclein protein (red) was seen in untreated in the *GBA1* mutant (N370S/WT) neurons. (M and N) The lower level of α-synuclein protein (red) was seen in the ambroxol-treated *GBA1* mutant (N370S/WT) neurons. The neuronal marker β-III tubulin was stained green. Nuclei were stained with DAPI (blue). Data are presented as means ± SEM. All comparisons were carried out with one-way ANOVA and Student's t test. ^∗∗^p < 0.01; ^∗^p < 0.05; NS, not significant (p > 0.05). Scale bars, 10 μm.

Ambroxol is a GCase small-molecule chaperone that has been reported to increase the expression of the *TFEB* transcription factor (McNeill et al., 2014). Ambroxol was incubated for 6 days with 40-day differentiated dopaminergic neurons derived from haNCSCs and induced a significant increase in GCase activity (CTRL 34.8% [p = 0.164], *GBA1* mutation 70.6% [p = 0.0004], and iPD 28.6% [p = 0.016]) and protein levels (CTRL 65.2% [p = 0.026], *GBA1* mutation 330.2% [p = 0.009], and iPD 190.7% [p = 0.050]) in all tested lines (Figures 6A, 6B, and 6D). Any contribution from non-lysosomal GCase (*GBA*2) was identified by using the inhibitor conduritol-B-epoxide (CBE) (Figure 6B). CBE inhibition of the haNCSC-derived neuronal cell lines led to 90% GCase inhibition in all cell lines. There was no significant change in hexosaminidase activities after ambroxol exposure (Figure 6C).

### Ambroxol Reduces α-Synuclein Levels in *GBA1* Mutant Neurons

A significant increase in α-synuclein protein levels was found in 40-day-old *GBA1* mutation-positive haNCSC-derived neurons carrying the N370S allele compared with controls (Figure 4F). Following ambroxol treatment, there was a significant reduction in α-synuclein levels in the *GBA1* mutation neurons (by 83%, p = 0.025). α-Synuclein levels were also reduced in control and iPD neurons, but these did not reach significance (35% [p = 0.316] and 39% [p = 0.267], respectively) (Figures 6E and 6F). α-Synuclein mRNA levels were not affected in any of the neuronal lines before or after ambroxol treatment (Figure 6G). Immunochemical staining of α-synuclein in untreated N370S mutant neurons (Figures 6K and 6L) and treated N370S mutant neurons (Figures 6M and 6N) indicated that ambroxol reduced α-synuclein levels in neurons.

Because it has been reported that cathepsin D is important in the degradation of α-synuclein (Sevlever et al., 2008), we investigated whether ambroxol modulated the level of this protein in the patient-derived cells. We found that the level of mature cathepsin D protein in *GBA1* mutant (N370S/WT) neurons increased following ambroxol treatment (Figures 6I and 6J).

### Ambroxol Treatment Upregulates Macroautophagy and Rescues Autophagy

**Figure 7:**
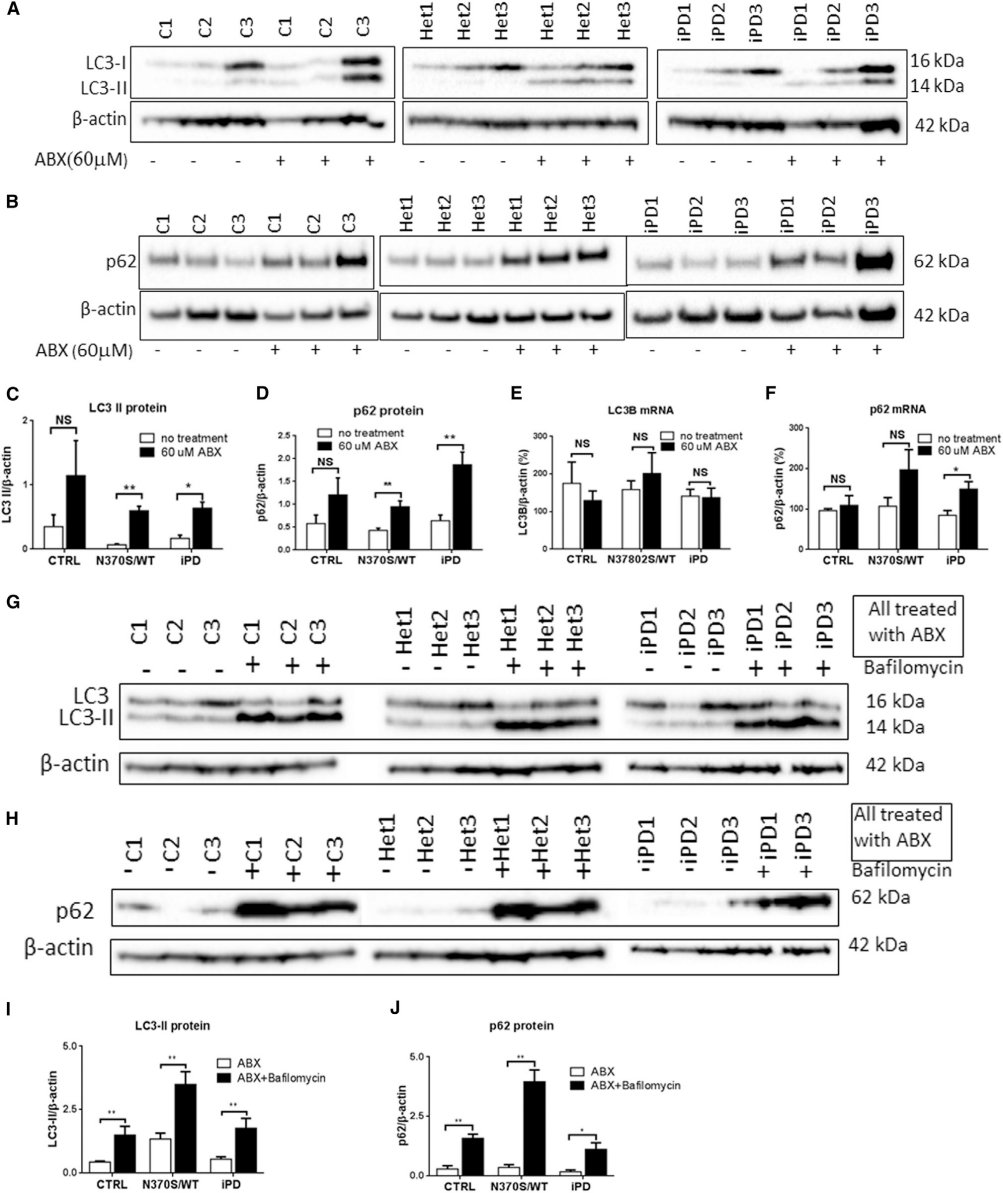
Ambroxol Treatment Upregulates Macroautophagy and Rescues the Autophagic Defect (A–D) Following ambroxol (ABX) treatment, levels of the autophagosome markers LC3-II and p62 were significantly increased in *GBA1* mutation (Het1–3; N370S/wt) and iPD (iPD1–3) cell lines, but not in control (C1–3; CTRL) cells. (E) LC3B mRNA levels were not significantly different between treated and untreated groups. (F) p62 mRNA levels were increased in iPD neurons after ambroxol treatment, but the increase was not statistically significant in control and *GBA1* mutation cells. (G–J) When the macroautophagy pathway was inhibited with bafilomycin in the ambroxol-treated neurons, LC3-II and p62 levels were increased further, indicating that the fusion of lysosomes with autophagosomes is normal but the autophagosome number is increased by the ambroxol treatment in *GBA1* mutation and iPD groups. For all three groups n = 3 cell lines; data are presented as means ± SEM. All comparisons were carried out with one-way ANOVA and Student's t test. ^∗∗^p < 0.01; ^∗^p < 0.05; NS, not significant (p > 0.05).

Following 6 days of ambroxol treatment, there was an upregulation of macroautophagy with significant increases seen in both LC3-II (Figures 7A and 7C) and p62 (Figures 7B and 7D) protein levels in *GBA1* mutation-positive and iPD neurons. Incubation of differentiated neurons with bafilomycin for 4 hr caused a further increase in LC3-II and p62 protein in all lines (Figures 7G–7J), indicating that ambroxol-increased LC3-II and p62 was not due to blockade of the autophagosome-lysosome fusion pathway. LC3B mRNA levels were not significantly increased after ambroxol treatment (Figure 7E). p62 mRNA was significantly increased in the PD group after ambroxol treatment; there was a trend toward increased *GBA1* mutant neurons, but this did not reach significance (Figure 7F). Ambroxol did not affect hexosaminidase activity (Figure 6C) or levels of LAMP1 and LAMP2a (Figures S4A–S4D).

## Discussion

Our study describes the preparation and analysis of dopaminergic neurons from NCSCs isolated from human adipose tissue, their use in the investigation of the biochemical consequences of the *GBA1* N370S mutation, and the potential for the small-molecule GCase chaperone, ambroxol, to reverse these effects. The use of haNCSCs provides direct access to pluripotent stem cells and provides an alternative source to those derived from fibroblast lines for the creation of neuronal lines. The haNCSC approach has advantages in terms of ease of access to neural crest cells, no requirement to reprogram, retention of the intact host genome, simple protocols for neuronal differentiation, production of homogeneous colonies, and less cost.

We have used this technique to develop both haNCSCs and iPSCs from controls and patients with the N370S *GBA1* mutation that causes GD and increases the risk for PD. Cell lineages were differentiated into dopaminergic neurons and compared with data from iPSCs derived from fibroblasts from the same donors. Compared with controls, the N370S mutant lines from both sources showed reduced GCase activity and protein levels and increased α-synuclein levels. These results are in agreement with a previous study using PD-derived iPSCs (Schondorf et al., 2014). We also show that the small-molecule GCase chaperone ambroxol can rescue GCase activity and reduce α-synuclein levels in human neurons. A recent study (Mazzulli et al., 2016) used a non-inhibitory small modulator to activate GCase in synucleinopathy culture model and found that activation of GCase enhanced the clearance of pathological α-synuclein. These findings support the hypothesis that increasing GCase activity reduces α-synuclein levels in dopaminergic neurons, and has significant implications for the use of GCase chaperones as treatments to reduce α-synuclein in PD patients.

Our results also have several important implications for the future study of *GBA1* mutations in PD. The ability easily to generate dopaminergic neurons from stem cells derived directly from the host provides an important source for the study of the biochemical consequences of GCase deficiency. We show that the N370S mutation reduces GCase protein levels and activity, and is associated with elevated α-synuclein levels. These are the core biochemical features in the study of *GBA1* mutations in PD as expressed in PD brain (Gegg et al., 2012) and provide a model with which to test therapeutic interventions to manipulate the GCase-α-synuclein pathway.

We show that the GCase defect was associated with impaired autophagosome formation in the N370S *GBA1* mutation dopaminergic haNCSCs, in agreement with Schondorf et al. (2014). We have shown that ambroxol rescues the biochemical abnormalities resulting from the N370S mutation. Ambroxol increased GCase activity in GD and PD fibroblasts and reduced α-synuclein levels in SHSY-5Y cells (McNeill et al., 2014). Ambroxol has also been shown to activate transcription factor EB and therefore to induce transcriptional upregulation of the CLEAR (coordinated lysosomal expression and regulation) network and saposin C levels (Ambrosi et al., 2015, McNeill et al., 2014). In the dopaminergic neuronal model derived from haNCSCs, ambroxol significantly increased GCase protein in the idiopathic PD and N370S *GBA1* mutant lines. There were parallel increases in activity in the N370S and iPD lines, with a trend in the controls that did not reach significance. We also found that ambroxol can significantly increase mature cathepsin D protein in N370S *GBA1* mutant lines. This may be a consequence of the transcriptional effects of ambroxol and may contribute to the mechanisms by which this drug reduces α-synuclein levels. An alternative or additional mechanism for the reduction in α-synuclein may be an increase in cathepsin D due to elevated GCase and ceramide levels induced by ambroxol.

Autophagy is considered to be a dynamic process comprising three sequential steps: formation of autophagosomes, the fusion of autophagosomes with lysosomes, and degradation. During this process, cytosolic form of LC3 is conjugated with phosphatidylethanolamine to form LC3-II, which is recruited to autophagosome membranes. p62 is a ubiquitin-binding scaffold protein acting as a cargo receptor, which is recruited in the formation of autophagosomes (Itakura and Mizushima, 2011). Ambroxol treatment significantly increased the steady-state levels of both LC3-II and p62 proteins in our model, suggesting that ambroxol increases autophagy by promoting autophagosome formation and turnover. This interpretation is further supported by a substantial increase in p62 mRNA. The unaltered LC3B gene expression suggests that the influence of ambroxol on LC3 synthesis is minimal.

The autophagic pathway has been increasingly implicated in a number of neurodegenerative diseases including PD (Mizushima et al., 2008), and the deregulation of autophagy is evident in the brains of PD patients (Levy et al., 2009). Autophagy has three different pathways, namely macroautophagy, microautophagy, and CMA. Previous studies have found that both macroautophagy and CMA pathways contribute to α-synuclein degradation in primary postnatal ventral midbrain neurons (Xilouri et al., 2008). Directly applying bafilomycin A1, an inhibitor of autophagy, to the cortex of α-synuclein transgenic mice increased α-synuclein levels in brain (Ebrahimi-Fakhari et al., 2012), suggesting that the autophagic pathway plays an important role in degradation of α-synuclein in neurons. Ambroxol treatment restores autophagosome formation in *GBA1* mutant lines, which in turn would enhance the degradation of α-synuclein and contribute to the decreased α-synuclein levels in the *GBA1* mutant lines. These results are important in confirming in patient-derived dopaminergic neuronal cells that ambroxol can rescue defects due to GCase deficiency, and appears to do so by several mechanisms. The reduction in neuronal α-synuclein levels offers the potential for ambroxol or similar drugs to be tried as a disease-modifying therapy in PD patients with *GBA1* mutations (Schapira and Gegg, 2013). The strategy to reduce α-synuclein in PD is common to a number of approaches to slow progression in PD (Schapira et al., 2014). Ambroxol is currently under investigation to confirm blood-brain barrier penetration before use in a larger trial. However, the haNCSC dopaminergic model used here provides the opportunity to test similar drugs to manipulate the GCase pathway for neuroprotection in PD.

## Experimental Procedures

### Subjects and Sample Collection

Samples from ten subjects were used in the study; written informed consent was obtained before subcutaneous fat was collected. The protocols used for collection were approved by the Royal Free Research Ethics Committee (REC number 10/H0720/21). PD was diagnosed according to UK Brain Bank criteria (Litvan et al., 2003). Differences in age and sex between groups were checked using one-way ANOVA. Skin-punch biopsies containing subcutaneous adipose tissues (4 mm in diameter) were obtained from arm of participants; the subcutaneous adipose tissues were dissected from skin and used for cell isolation. Ten subjects were divided into three groups according to their genotype (wt/wt healthy, N370S/wt, and wt/wt iPD). In some of the experiments we used four heterozygous mutation lines including three heterozygous carriers and one heterozygous PD. This was initially to examine whether there were differences between these cell lines in terms of changes associated with the *GBA1* mutation. We examined GCase activity, SNCA, and LAMP1 protein level in all of these four cell lines before and after ambroxol treatment, and found that the changes in these four cell lines are all similar; we have not observed any significant differences between heterozygous carriers and heterozygous PD cell lines. Therefore, we used three heterozygous carriers for the remainder of the experiments. More detailed information regarding subjects can be found in Table S1.

### Isolation and Maintenance of haNCSCs

Human subcutaneous adipose tissues were washed once with growth medium and dissected into small pieces (less than 0.2 cm diameter). The small pieces of adipose tissues were explanted in fibronectin-coated 6-well plates. A sterilized coverslip was used to cover the tissue in order to prevent tissue floating in the medium. Media were changed every 4 days until migrated cells reached 60%–70% confluency. The cells were dissociated from the well with Accutase (Millipore) and seeded in normal culture dishes with growth medium. Cells were passaged with a ratio of 1:3 when they reached 70%–80% confluency. At this density, the growth medium was removed; cells were washed with PBS without Ca^2+^ and were incubated with Accutase (1 mL/dish; 100 × 20 mm) at 37°C for 3–5 min. Growth medium was added to the dissociated cells and the medium containing cells was divided over three individual dishes. Cells were cultured and maintained in growth medium at 37°C with 5% CO_2_/95% air.

### Neurosphere Formation and Maintenance

When haNCSC reached to 70%–80% confluency, cells were dissociated with Accutase at 37°C for 3–5 min. Growth medium was added to the dish to inhibit Accutase activity. Cells were collected and pelleted by centrifugation at 200 × *g* for 10 min. The cell pellet was resuspended with neurosphere formation medium, transferred to an uncoated 12-well plate, and incubated at 37°C with 5% CO_2_/95% air. Neurospheres formed within 24 hr in neurosphere formation medium. The neurospheres were cultured and maintained in the same conditions with medium changes every 5 days.

### Dopaminergic Neuronal Differentiation of haNCSCs with Co-culture System

PA6 cells were maintained as described previously (Kawasaki et al., 2000). For differentiation experiments, PA6 cells were seeded on fibronectin-coated chamber slides 1 day before introducing haNCSCs into the culture. haNCSCs were harvested and resuspended in neurobasal medium supplemented with B27 (1×). The medium was removed from the chamber and PA6 cells were washed with PBS once. haNCSC cells in neurobasal medium supplemented with B27 were introduced into the chamber with a density of 200 cells/chamber. Medium was changed every 2 days up to 40 days.

### Dopaminergic Neuronal Differentiation with Defined Medium and Quantification

haNCSC were detached with Accutase solution and the Accutase was neutralized by the addition of growth medium. Cells were seeded in fibronectin-coated 6-well plates at a density of 2.4 × 10^4^ cells/well (for immunocytochemistry assay, cells were seeded onto coverslips coated with fibronectin within a 6-well plate) with growth medium. After 24 hr of seeding, the growth medium was removed from the well; cells were washed once with neurobasal medium and then cultured with neuronal induction medium. The cells were cultured with 5% CO_2_/95% air for 10 days for neuronal induction. Following neuronal induction, the neuronal induction medium was replaced with neuronal maturation medium. The cells were cultured with 5% CO_2_/95% air for 30 days. The volume of neuronal maturation medium was 1.7–2 mL/well (6-well plate). The medium was changed with the freshly made neuronal maturation medium every 8 days during maturation. For the purposes of assessing the percentage of dopaminergic neurons in the differentiated cells, the number of total cells and TH-positive immunostaining neurons were counted. For the three groups each group contained three subjects, three slides of each subject were studied, and three fields of each slide were used for counting. The percentage of dopaminergic neurons was then calculated according to the counts.

### Ambroxol Treatment of haNCSC-Derived Neurons

haNCSC-derived neuronal cultures were treated at day 40 of neuronal differentiation with ambroxol (A9797, Sigma-Aldrich) on alternate days for 6 days. Ambroxol was dissolved in DMSO and further diluted in cell culture medium to give a final concentration of 60 μM. Controls were cultured with medium containing DMSO instead of ambroxol in DMSO. At day 6, the cells were washed twice with PBS prior to harvest. Cell pellets were frozen at −80°C for storage.

### Statistical Analysis

Data are expressed as mean ± SEM and statistical significance between groups was determined by one-way ANOVA followed by the two-tailed t test. A p value of <0.05 was considered significantly different. All data were analyzed by GraphPad Prism 6 statistical software.

## Author Contributions

S.-Y.Y. designed research studies, conducted experiments, acquired and analyzed data, and reviewed the manuscript. M.B. designed research studies, conducted experiments, acquired and analyzed data, and reviewed the manuscript. K.-Y.C. conducted some experiments, acquired and analyzed data, and reviewed the manuscript. J.-W.T. analyzed data and reviewed the manuscript. A.H.V.S. designed research studies, analyzed data, obtained funding, and wrote the manuscript.

## Supplemental Information

Document S1. Supplemental Experimental Procedures, Figures S1–S4, and Tables S1–S3

Document S2. Article plus Supplemental Information
