# Supplementary material for: A Human Neural Crest Stem Cell-Derived Dopaminergic Neuronal Model Recapitulates Biochemical Abnormalities in GBA1 Mutation Carriers
Source: Stem Cell Reports. 2017 Feb 16;8(3):728–42. doi: 10.1016/j.stemcr.2017.01.011 (PMC5355624; doi:10.1016/j.stemcr.2017.01.011)
Supplement: Document S1. Supplemental Experimental Procedures, Figures S1–S4, and Tables S1–S3 [file mmc1.pdf]

Stem Cell Reports, Volume *8*

Supplemental Information

A Human Neural Crest Stem Cell-Derived Dopaminergic Neuronal Model Recapitulates Biochemical Abnormalities in *GBA1* Mutation Carriers

Shi-Yu Yang, Michelle Beavan, Kai-Yin Chau, Jan-Willem Taanman, and Anthony H.V. Schapira

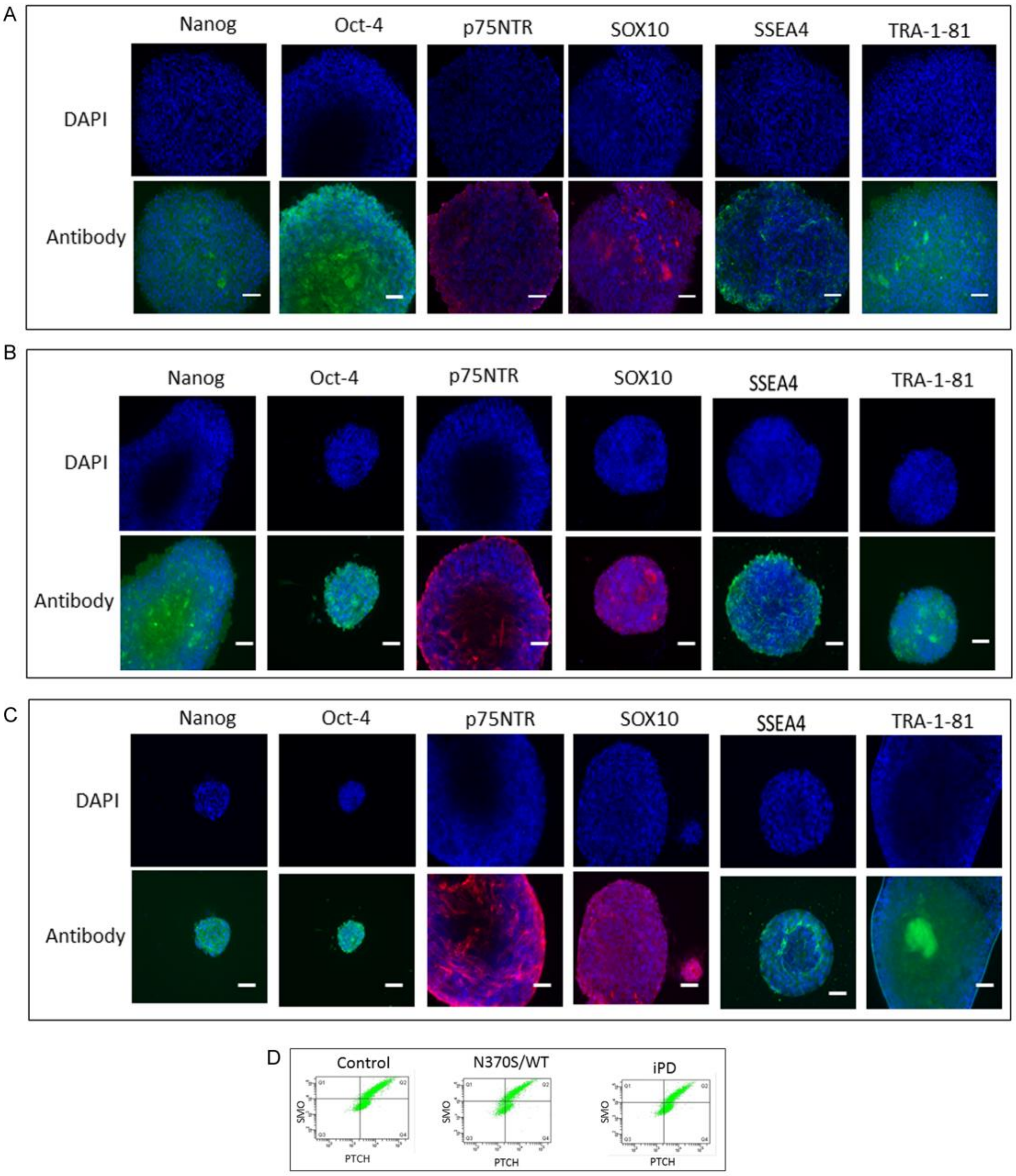

Figure S1 (related to Fig 1) Comparison of pluripotent properties between adipose neural crest stem cells derived from control, GBA mutant and iPD subjects.

To examine whether there are differences in the pluripotent properties of adipose neural crest stem cells derived from control, carriers of a *GBA1* mutation and iPD patients, the expression of pluripotency genes (*NANOG, OCT4, SSEA4, TRA-1-81*, all green) and the neural crest genes (*p75NTR* and *SOX10*, all red) were examined with immunochemistry. All cells derived from control (A), heterozygous mutant *GBA1* (B) and iPD (C) subjects express pluripotency and neural crest genes. There was no noticeable difference in the expression of these genes between control, heterozygous mutant *GBA1* and iPD subjects. The expression of sonic hedgehog (a signalling molecule involved in the development of early neural crest progenitors) was examined by FACS analysis. There was no obvious difference in the expression of sonic hedgehog between control, heterozygous mutant *GBA1* and iPD subjects (D). Nuclei were stained with DAPI (blue). (Bars: 50 mm)

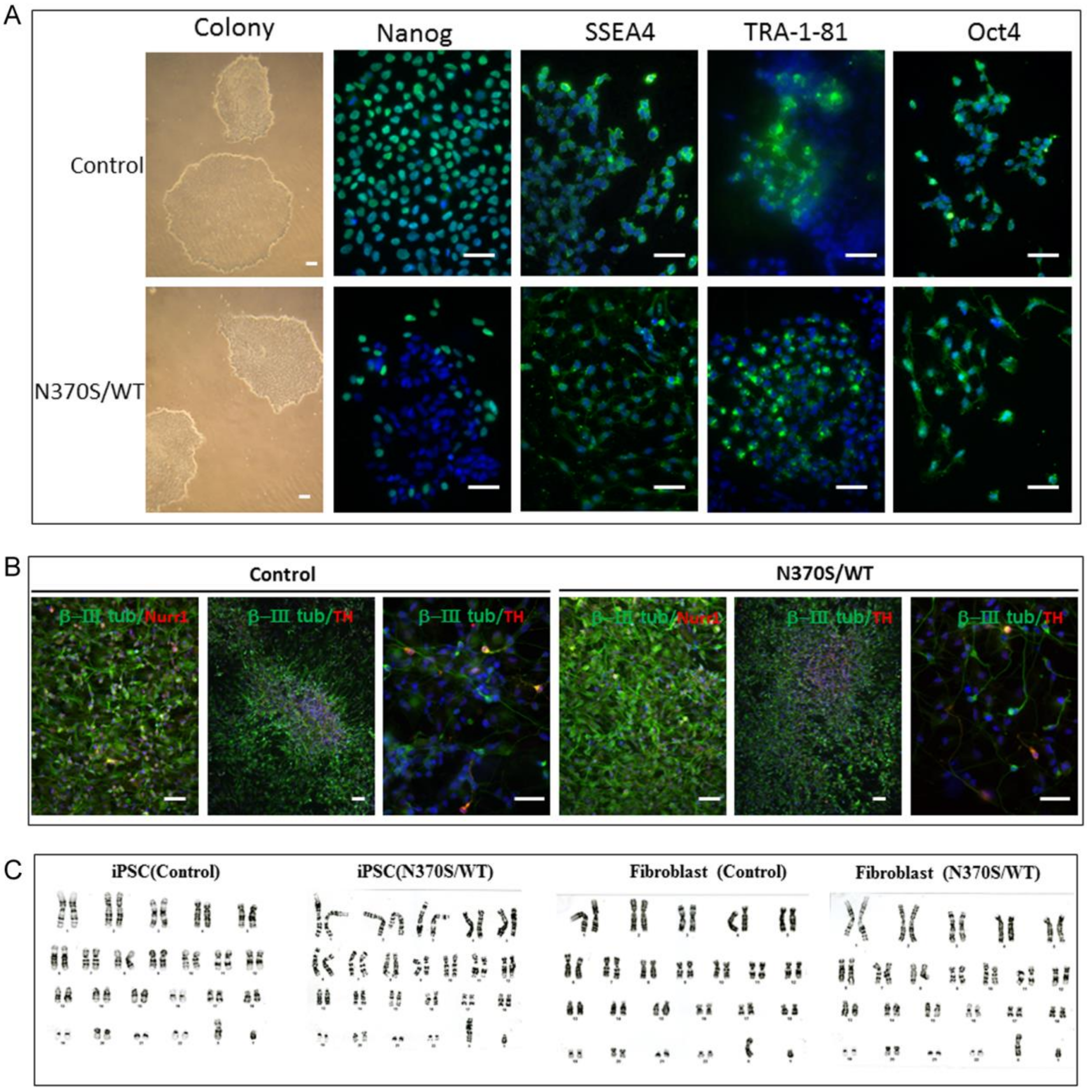

Figure S2 (related to Fig 3)
The iPSC clones from both control and mutant subjects expressed pluripotency genes (*NANOG, SSEA4, TRA-1-81* and *OCT4*, all green) (A). The iPSC stem cells were differentiated into dopaminergic neurons which expressed the neuronal marker, b-III tubulin (b-III tubulin, green) and the dopaminergic neuronal markers tyrosine hydroxylase (TH, red) and nuclear receptor related protein 1 (NURR1, red) (B). Karyotyping (C) was performed to examine the integrity of the chromosomes in fibroblast and iPSC derived from control subjects and subjects with the *GBA1* mutation. Nuclei were stained with DAPI (Blue). (Bars: 50 mm)

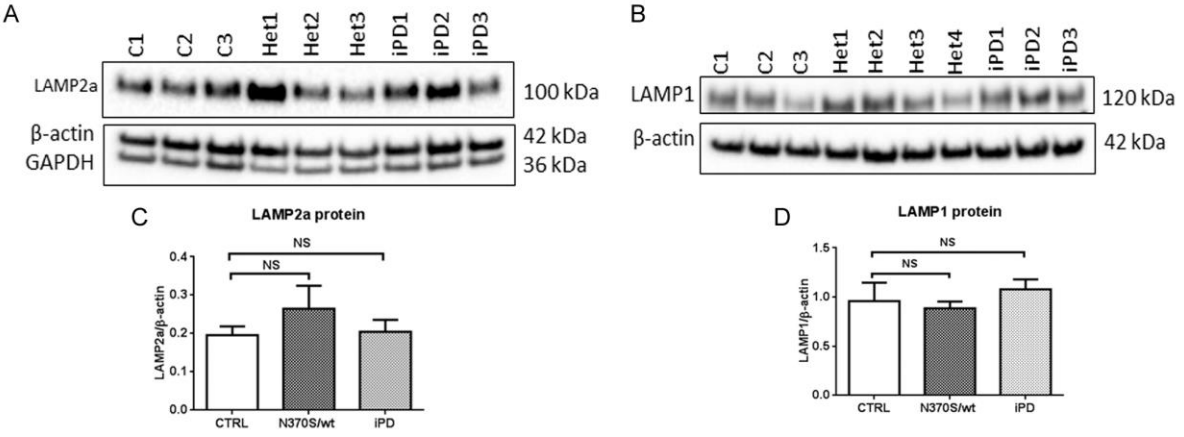

Figure S3 (related to Fig 5)

The levels of lysosomal proteins such as LAMP2a (A and C) and LAMP1 (B and D) were not significantly different between the 3 groups. This suggests that the lysosomal content is not affected by the *GBA 1* mutation in human neuronal cells. For all groups n=3-4 cell lines, error bars reflect SEM. All comparisons were carried out with one-way ANOVA and Student's t-test NS: $p>0.05$.

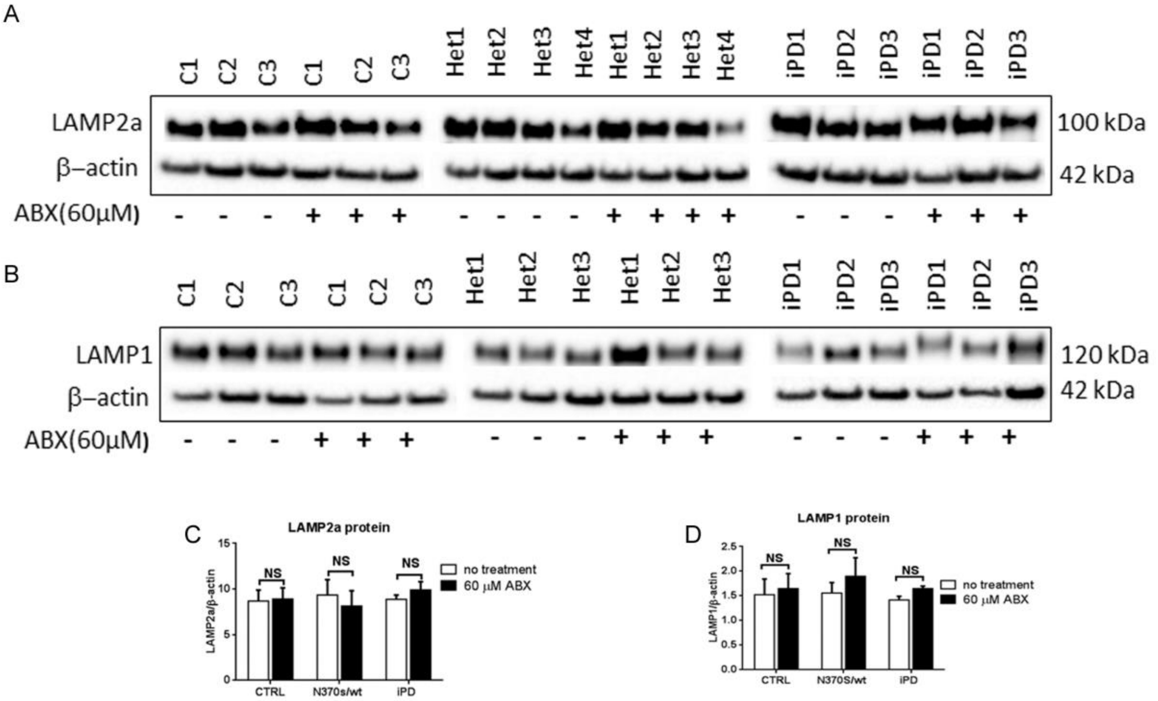

Figure S4 (related to Fig 5)
The level of the lysosomal proteins LAMP2a (A and C) and LAMP1 (B and D) was not affected by ambroxol treatment in the three groups, suggesting that ambroxol treatment is not causing an overall expansion of lysosomal compartment. For all groups n=3-4 (cell lines), error bars reflect SEM. All comparisons were carried out with one-way ANOVA and Student's t-test NS: $p>0.05$.

Table S1 **Summary of patient-derived adipose neural crest stem cells**

Subject	Genotype	Gender	Diagnosis	Age at biopsy
C1	wt/wt	Female	CTRL	67
C2	wt/wt	Female	CTRL	70
C3	wt/wt	Male	CTRL	81
Het1	N370S/wt	Female	*GBA* Carrier	82
Het2	N370S/wt	Male	*GBA* Carrier	52
Het3	N370S/wt	Female	*GBA* Carrier	67
Het4	N370S/wt	Male	GBA-PD	80
PD1	wt/wt	Male	iPD	80
PD2	wt/wt	Female	iPD	75
PD3	wt/wt	Female	iPD	53

Table S2 **List of antibodies**

Antibody	Dilution	Application	Source	Cat. No.
P75NGF R	1:500	ICC	Abcam	ab8874
SOX 10	1:250	ICC	Santa Cruz	sc17342
Nestin	1:500	ICC	Millipore	mab5326
Fatty Acid-binding protein 4	1:100	ICC	R &D system	af3150
SSEA-4	1:100	ICC	Abcam	Ab16287
Nanog	1:500	ICC	Abcam	Ab17336
OCT-4	1:250	ICC	Millipore	MAB4401
LMX1	1:250	ICC	Millipore	MAB10533
PTCH	1:500	FACS	Abcam	Ab129341
SMO	1:50	FACS	Millipore	MAB360
Beta III Tubulin	1:500	WB/ICC	Abcam	ab18207
Tyrosine Hydroxylase	1:1000	WB/ICC	Abcam	ab6211
Nurr1	1:200	ICC	Abcam	ab55769
Dopamine transporter	1:1000	ICC	Millipore	Ab5802
GBA	1:1500	WB	Calbiochem	AP1140
β-ACTIN	1:5000	WB	Abcam	ab8227
BiP	1:1000	WB	Abcam	ab21685
LAMP1	1:1000	WB	Abcam	Ab24170
LAMP2a	1:500	WB	Abcam	ab18528
Hsc70	1:1000	WB	Abcam	ab51052
GAPDH	1:5000	WB	Abcam	ab9485
LC3	1:1000	WB	Cell Signaling	2775
p62	1:1500	WB	BD Biosciences	610832
Alpha-synuclein	1:500	WB/ICC	Abcam	ab1903

Table S3 **List of PCR primers**

Pluripotent genes	Forward/reverse primers	Sequences of Primers
SOX2	Forward	GCTACAGCATGATGCAGGACCA
	Reverse	TCTGCGAGCTGGTCATGGAGTT
Oct4	Forward	CCTGAAGCAGAAGAGGATCACC
	Reverse	AAAGCGGCAGATGGTCGTTTGG
Nanog	Forward	CTCCAACATCCTGAACCTCAGC
	Reverse	CGTCACACCATTGCTATTCTTCG
Rex1	Forward	GGAAATAGCAGAGTGCTTCGC
	Reverse	CTCTTCTGTTCTGTTCACACAG
cMYC	Forward	CTGAGACAGATCAGCAACAACC
	Reverse	TCTTTTATGCCCAAAGTCCAAT
KLF4	Forward	CATCTCAAGGCACACCTGCGAA
	Reverse	TCGGTCGCATTTTTGGCACTGG

Supplemental Experimental Procedures

***GBA1* Screening**: *GBA1* mutation status in all participants was confirmed by sequencing exons 1 to 11 of the *GBA1* gene, using a published protocol (Neumann et al. 2009)with polymerase chain reaction (PCR) primers designed exclusively for regions of the *GBA1* gene not found in the pseudogene. After amplification by PCR the product was run on a 1% agarose gel with ethidium bromide and size-checked to ensure it was not the pseudogene. Sanger sequencing was performed for each exon and flanking intronic sequences using the Dye Terminator Sequencing Kit (Applied Bio-systems) on an ABI 3700xl genetic analyser.

Growth medium: DMEM, (High Glucose, Gutamax, Life technologies) supplemented with foetal bovine serum (10%), Sodium Pyruvate (1 mM), Uridine (50 μg/ml), Penicillin (50 units/ml), Streptomycin (50 μg/ml), Fungizone (Amphotericin B, 1.25 μg/ml).

Neurosphere formation medium: DMED/F12 (1:1, Glutamax, Life technologies) supplemented with B27 supplement (1x, Life technologies), recombinant human FGF basic (146 aa) (20 ng/ml, R&D Systems), recombinant human EGF (10 ng/ml, AbD Serotec), recombinant human leukemia inhibitory factor (10 ng/ml, Sigma), Penicillin (50 units/ml), Streptomycin (50 μg/ml),Fungizone (1.25 μg/ml).

Neuronal induction medium (first 10 days of differentiation): Neurobasal medium supplemented with B-27 supplement (1x), recombinant Human Sonic Hedgehog (250 ng/ml, R&D Systems), recombinant Human/Mouse FGF-8b (100 ng/ml), Recombinant Human FGF basic (50 ng/ml), Penicillin (50 units/ml), Streptomycin (50 µg/ml),Fungizone (1.25 µg/ml).

Neuronal maturation medium: (11-40 days of differentiation): Neuronal induction medium further supplemented with recombinant Human/Mouse/Rat/Canine/Equine BDNF (50 ng/ml, R&D Systems).

Flow cytometry: Cells were detached with accutase solution, washed with PBS containing 1% foetal bovine serum and pelleted by centrifuging for 10 minutes at 200 × *g*. Cells were fixed with 2% paraformaldehyde for 30 minutes on ice and incubated with anti-smoothened (SMO) and anti-patched (PTCH) antibodies on ice for 30 minutes. Following washing secondary antibodies were incubated on ice for 30 minutes. Cells were immediately analysed by BD FACScan. BD CellQuest software (BD Biosciences) was used for analyses. Primary antibody isotype control and unlabelled cells (no primary and secondary antibodies) were used for FACS gating.

iPSCs generation and dopaminergic neuronal differentiation: Yamanaka plasmids were purchased from Addgene. Plasmids including pCXLE h*Oct4 shp53-F*, *pCXLE hSK* and *pCXLE hUL* have been previously described (Okita et al. 2011). Early passage fibroblasts (less than 12 passages) in logarithmic growth phase were used for iPSC generation. Fibroblasts were harvested; the cell pellet was washed once with PBS and suspended in resuspension buffer (Neon Transfection System, Invitrogen). 10^6 cells were transfected with 1 µg DNA of each plasmid using the Neon transfection system (Invitrogen). 2×10^5 viable cells were transferred into one well of a 6 well plate coated with hES cell qualified Matrigel with growth medium. After 24 hours of transfection, growth medium was replaced with mTESR1 (StemCellTechnologies) medium supplemented with sodium butyrate (0.5 mM). Following 12 days of transfection, mTESR1 medium alone was used. Medium was changed daily for the whole period of iPSC generation. After 30 days of transfection, the whole well was stained with TRA-1-81 antibody and positive colonies were selected and isolated for further characterisation and differentiation.

iPSCs were differentiated into dopaminergic neurons with a midbrain dopaminergic neuronal induction method (Kriks et al. 2011; Stanslowsky et al. 2014).

Karyotyping: Karyotyping was performed by full G-band analysis of 20 cells in 3 human iPSC cultures (1x wild type, 1x heterozygous *GBA1* mutation carrier, and 1x iPD) to examine the integrity of the chromosomes by The Doctor's Laboratory at Whitfield Street, London. All cells tested showed a normal banding pattern.

Immunochemistry: Cells were washed with PBS two times, each wash lasting 5 minutes. Cells were fixed with 4% paraformaldehyde for 15 minutes at room temperature and subsequently permeabilized with 0.25% Triton X-100 for 15 minutes. Following three washings, cells were blocked with 10% goat serum in PBS for 30 minutes and incubated with primary antibodies overnight at 4°C. Appropriate secondary antibodies conjugated with fluorescein were used to visualize the primary antibodies. Details of primary and secondary antibodies and their dilution can be found in supplementary table 2.

Lysosomal enzymatic activity assays: Cell pellets were re-suspended in water and sonicated in a water-bath sonicator for 1 minute. GCase activity was determined in cell lysates of about 1 µg protein with a previous reported method (Cleeter et al. 2013).

Western blotting: Cells were harvested, washed with PBS, and processed as previously described (McNeill et al. 2014) . Proteins were extracted using urea/SDS buffer (8 M urea, 2% SDS, 10 mM Tris·HCl pH 6.8) containing protease inhibitors. To limit the effect of DNA content in the protein solution, the extraction was incubated with DNase at 37° C for 1 hour to remove DNA. In total, 40 µg of protein was loaded on a 4 – 12% Bis-Tris Gel (NuPAGE, Invitrogen) and transferred to a polyvinylidene difluoride membrane (Millipore). The membrane was incubated with primary antibodies in 5% milk, PBS, 0.4%Tween 20 at room temperature for 2 hours. Following 3 washing, the membranes were incubated with the appropriate secondary antibodies conjugated to horseradish peroxidase for 1 hour at room temperature. The bands were visualized with Clarity ECL Western Blotting Substrate (Bio-Rad). Analyses were carried out

using Image Lab software (BioRad). The details of primary and secondary antibodies and their dilution can be found in supplemental table 2. .

Autophagy studies: Where indicated, cells were treated with 0.2 µM bafilomycin (EMD, Millipore) for 4 hours at 37 °C in the presence of 5% CO_2, and then fixed or lysed for western blot analysis. LC3/LC3-II and p62 levels were quantified using Image Lab software and normalized for β-actin. The LC3 flux was quantified by dividing levels of LC3-II after bafilomycin treatment for 6 h by the level of LC3-II without treatment.

Real time reverse transcriptase quantitative PCR (RT-qPCR): Total RNA was extracted from cell pellets with the RNeasy mMni Kit with on-column DNase digestion according to the manufacture's guidelines (Qiagen). RNA yield and quality were assessed by Nanodop UV spectrophotometry. cDNA was generated from 1.0 µg of RNA with the Quantitect Reverse Transcription Kit from Qiagen. All RT-qPCR reactions (except for p62 and LC3B) were performed using the power SYBR Green PCR master mix (Life Technologies) and assayed with the Step One Real Time PCR system (Applied Bio-system). For p62 and LC3B, qPCR reaction was performed using TaqMan Gene Expression Assays (Life Technologies; Hs01061917_g1 for human sequesosome 1 p62, Hs00797944s1 for human microtubule-associated protein 1 light chain 3 beta, Hs99999903m1 for human beta-actin) in the StepOne Real-Time PCR Systems (Life Technologies). Fold-change in gene expression was calculated using the $2^{-\Delta\Delta CT}$method, based on biological reference samples and beta-actin messenger RNA levels for normalization. All the results were obtained from the evaluation of two technical duplicates of three independent experiments.

Supplemental References

Cleeter, M.W., Chau, K.Y., Gluck, C., Mehta, A., Hughes, D.A., Duchen, M., Wood, N.W., Hardy, J., Mark, C.J., & Schapira, A.H. 2013. Glucocerebrosidase inhibition causes mitochondrial dysfunction and free radical damage. *Neurochem.Int.*, 62, (1) 1-7

Kriks, S., Shim, J.W., Piao, J., Ganat, Y.M., Wakeman, D.R., Xie, Z., Carrillo-Reid, L., Auyeung, G., Antonacci, C., Buch, A., Yang, L., Beal, M.F., Surmeier, D.J., Kordower, J.H., Tabar, V., & Studer, L. 2011. Dopamine neurons derived from human ES cells efficiently engraft in animal models of Parkinson's disease. *Nature*, 480, (7378) 547-551

McNeill, A., Magalhaes, J., Shen, C., Chau, K.Y., Hughes, D., Mehta, A., Foltynie, T., Cooper, J.M., Abramov, A.Y., Gegg, M., & Schapira, A.H. 2014. Ambroxol improves lysosomal biochemistry in glucocerebrosidase mutation-linked Parkinson disease cells. *Brain*, 137, (Pt 5) 1481-1495

Neumann, J., Bras, J., Deas, E., O'Sullivan, S.S., Parkkinen, L., Lachmann, R.H., Li, A., Holton, J., Guerreiro, R., Paudel, R., Segarane, B., Singleton, A., Lees, A., Hardy, J., Houlden, H., Revesz, T., & Wood, N.W. 2009. Glucocerebrosidase mutations in clinical and pathologically proven Parkinson's disease. *Brain*, 132, (Pt 7) 1783-1794

Okita, K., Matsumura, Y., Sato, Y., Okada, A., Morizane, A., Okamoto, S., Hong, H., Nakagawa, M., Tanabe, K., Tezuka, K., Shibata, T., Kunisada, T., Takahashi, M., Takahashi, J., Saji, H., & Yamanaka, S. 2011. A more efficient method to generate integration-free human iPS cells. *Nat.Methods*, 8, (5) 409-412

Stanslowsky, N., Haase, A., Martin, U., Naujock, M., Leffler, A., Dengler, R., & Wegner, F. 2014. Functional differentiation of midbrain neurons from human cord blood-derived induced pluripotent stem cells. *Stem Cell Res.Ther.*, 5, (2) 35
